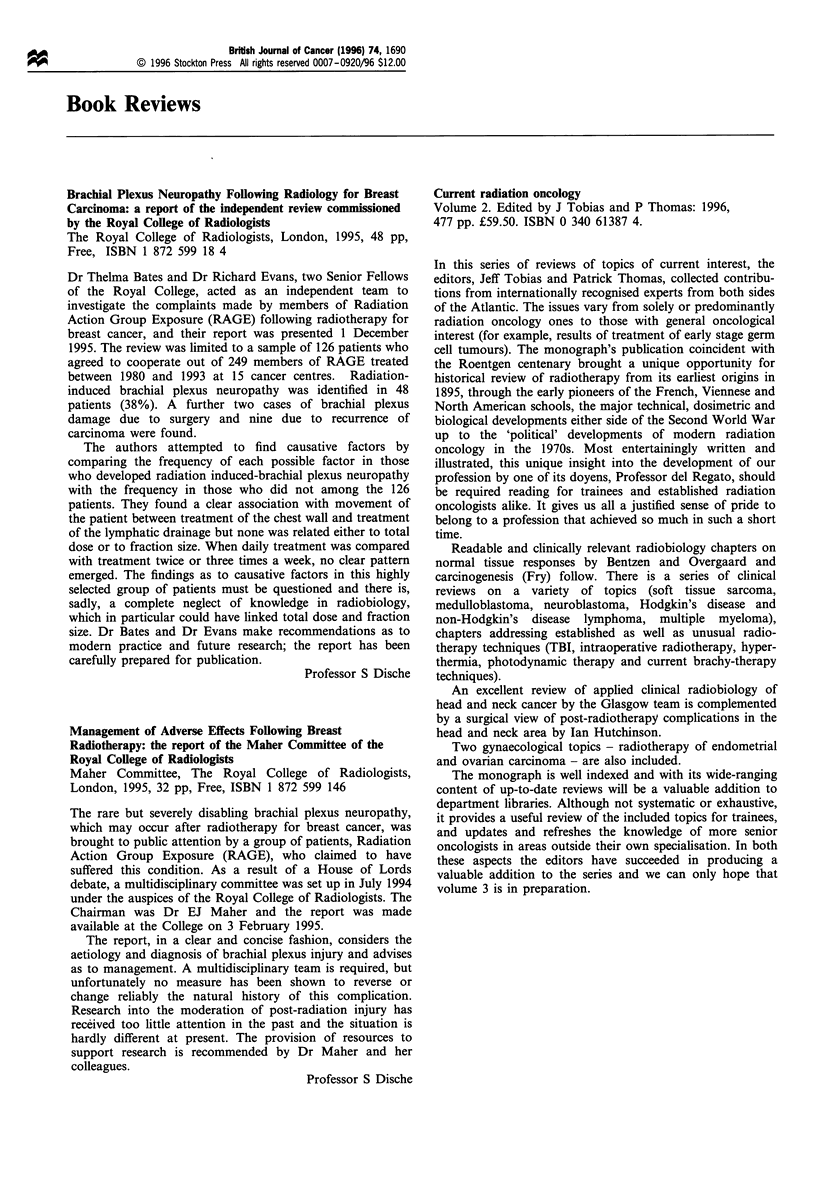# Current radiation oncology

**Published:** 1996-11

**Authors:** 


**Current radiation oncology**
Volume 2. Edited by J Tobias and P Thomas: 1996, 477 pp. £59.50. ISBN 0 340 61387 4.

In this series of reviews of topics of current interest, the editors, Jeff Tobias and Patrick Thomas, collected contributions from internationally recognised experts from both sides of the Atlantic. The issues vary from solely or predominantly radiation oncology ones to those with general oncological interest (for example, results of treatment of early stage germ cell tumours). The monograph's publication coincident with the Roentgen centenary brought a unique opportunity for historical review of radiotherapy from its earliest origins in 1895, through the early pioneers of the French, Viennese and North American schools, the major technical, dosimetric and biological developments either side of the Second World War up to the 'political' developments of modern radiation oncology in the 1970s. Most entertainingly written and illustrated, this unique insight into the development of our profession by one of its doyens, Professor del Regato, should be required reading for trainees and established radiation oncologists alike. It gives us all a justified sense of pride to belong to a profession that achieved so much in such a short time.

Readable and clinically relevant radiobiology chapters on normal tissue responses by Bentzen and Overgaard and carcinogenesis (Fry) follow. There is a series of clinical reviews on a variety of topics (soft tissue sarcoma, medulloblastoma, neuroblastoma, Hodgkin's disease and non-Hodgkin's disease lymphoma, multiple myeloma), chapters addressing established as well as unusual radiotherapy techniques (TBI, intraoperative radiotherapy, hyperthermia, photodynamic therapy and current brachy-therapy techniques).

An excellent review of applied clinical radiobiology of head and neck cancer by the Glasgow team is complemented by a surgical view of post-radiotherapy complications in the head and neck area by Ian Hutchinson.

Two gynaecological topics – radiotherapy of endometrial and ovarian carcinoma – are also included.

The monograph is well indexed and with its wide-ranging content of up-to-date reviews will be a valuable addition to department libraries. Although not systematic or exhaustive, it provides a useful review of the included topics for trainees, and updates and refreshes the knowledge of more senior oncologists in areas outside their own specialisation. In both these aspects the editors have succeeded in producing a valuable addition to the series and we can only hope that volume 3 is in preparation.